# Frequency and clinical impact of viraemia in paediatric patients undergoing therapy for cancer

**DOI:** 10.1038/s41598-024-65641-w

**Published:** 2024-06-27

**Authors:** Anke Barnbrock, Annemarie Berger, Melchior Lauten, Martin Demmert, Jan-Henning Klusmann, Sandra Ciesek, Konrad Bochennek, Thomas Lehrnbecher

**Affiliations:** 1 Department of Paediatrics, Division of Haematology, Oncology and Haemostaseology, Goethe University Frankfurt, Theodor-Stern-Kai 7, 60590 Frankfurt/Main, Germany; 2 Institute for Medical Virology, Goethe University Frankfurt, Frankfurt/Main, Germany; 3 Department of Paediatric Haematology and Oncology, Hospital for Children and Adolescents, University Hospital Schleswig-Holstein, Lübeck, Germany

**Keywords:** Child, Cancer, Viraemia, Febrile neutropenia, Medical research, Oncology

## Abstract

In contrast to transplant recipients, there is a paucity of data regarding frequency and clinical significance of viraemia in children receiving conventional chemotherapy. In a prospective observational study, we assessed the frequency of and clinical impact of viraemia with cytomegalovirus (CMV), Epstein-Barr virus (EBV), adenovirus, human herpesvirus-6 (HHV6) and herpes-simplex virus 1/2 (HSV1/2) in paediatric cancer patients at diagnosis, at a routine examination during intensive chemotherapy, and during febrile neutropenia (FN). Seventy-nine patients (median age 6 years; 66 children with haematological malignancies) were included in the study. Overall, 362 blood samples were analysed, 72 from the time at diagnosis (11.1% with positive PCR result), 118 during a regular control after chemotherapy (11.0% positive), and 159 during FN (8.8% positive). The overall positivity rate was 9.6% (CMV 3.3%, HHV6 2.7%, HSV 2.2%, EBV 0.8% and adenovirus 0.3%). There were no significant differences between FN episodes with and without viraemia in terms of duration of fever or neutropenia/lymphopenia, severity of mucositis (> II^0^), incidence of diarrhea and ICU admission. Our results indicate that viraemia in paediatric cancer patients generally does not have a major clinical impact, and may help in the decision regarding the indication of routine evaluation for viraemia in febrile neutropenic, but otherwise asymptomatic children.

## Introduction

Febrile neutropenia is a common infectious complication in children receiving chemotherapy for cancer and occurs in the majority of patients, often several times during the course of intensive treatment^1,2^. Children presenting with febrile neutropenia are usually hospitalised and are treated with broad-spectrum antibiotics^3^. Therefore, this infectious complication has a major impact on the patients´ and families´ quality of life as well as on resources of the health care system. Importantly, in only one third of febrile neutropenic patients, a bacterial organism can be isolated in the blood stream, whereas in the majority of patients, fever remains the only sign of the infection (“fever of unknown origin”, FUO)^4^.

Viral reactivation is a common problem in children and adults after haematopoietic cell transplantation (HCT), and in this setting, antiviral treatment is often indicated. Although the clinical course may be asymptomatic, severe, even fatal diseases are seen in a considerable number of patients. In a recent study in 404 allogeneic HCT recipients, more than 90% of the patients showed during the first 100 days after transplantation reactivation of at least one of the viruses studied, namely cytomegalovirus (CMV), Epstein-Barr virus (EBV), human herpesvirus-6 (HHV6), adenovirus and BK polyomavirus^5^. Age younger than 21 years was a risk factor for viraemia with multiple viruses, and increased virus detection was identified as a predictor of overall mortality after accounting for factors such as delayed immune reconstitution or severity of acute graft-versus-host disease.

It seems plausible that viraemia might also play a role in children receiving chemotherapy for cancer, but unfortunately, the incidence of viraemia in this setting is relatively unclear, as reported rates vary widely. In addition, most of the studies addressed the clinical relevance of a positive virus result, and therapy was instituted according to the physician´s discretion, leading to uncertainties in the implementation of therapeutic interventions. On the other hand, a positive virus result in the blood might help to optimise the time of empirical antibiotic therapy and hospitalisation in neutropenic pediatric cancer patients with fever, as it has been shown in a randomised study in febrile neutropenic children with viral respiratory infection and has been identified as a research gap in recently published pediatric specific guidelines^3,6^. We therefore determined in this prospective and blinded observational pilotstudy the frequency of viraemia with CMV, HHV6, herpes simplex virus (HSV1/2), EBV, and adenovirus in children receiving therapy for various malignancies and evaluated whether detection of viraemia during febrile neutropenia (FN) has an impact on the clinical course.

## Patients and methods

### Patients

All children and adolescents younger than 18 years of age treated for acute lymphoblastic or acute myeloid leukemia (ALL and AML), non-Hodgkin lymphoma (NHL), Ewing sarcoma, or neuroblastoma were eligible for this prospective, non-interventional observational study. Patients with relapsed malignancy and patients with inborn errors of immunity were not included. Patients were treated according to Berlin-Frankfurt-Münster (BFM)-based protocols (e.g., AIEOP-BFM ALL 2017 (EudraCT 2016–001,935-12), AML-BFM 2012 (EudraCT 2013–000,018-39), B-NHL 2013 (EudraCT 2013–003,253-21) or NHL-BFM Registry 2012 (https://www.gpoh.de/kinderkrebsinfo/content/health_professionals/ clinical_trials/general_information/index_eng.html; accessed November 10, 2023), EWING 2008 (EudraCT 2008–003,658-13), or the GPOH guidelines for the treatment of neuroblastic tumours^7^. None of the patients had received antiviral prophylaxis. All patients and/or caregivers provided written informed consent. The study was reviewed and approved by the local Ethical committee of the University of Frankfurt (19/402).

### Definitions

Fever was considered as temperature > 38.5 °C once or 38.0–38.5 °C twice within a 1-h interval, and neutropenia as an absolute neutrophil count < 500/μl. Lymphopenia was defined according to age-dependent reference values given by Tosato et al.^8^. Mucositis was measured using the Oral Assessment Guide (OAG)^9^. One cycle of chemotherapy was defined as the time from the start of chemotherapy until the day before the start of the next cycle of chemotherapy.

### Study design

Clinical and laboratory data were collected throughout intensive chemotherapy, and the viral load for CMV, HSV1/2, HHV6, EBV, and adenovirus was assessed in the plasma at several time points. First, the viral load was evaluated at the time of diagnosis of the malignancy. At that time, also specific antibody titres against CMV, HSV1/2, and EBV were determined, whereas antibody titres for HHV6 and adenovirus were not regularly assessed. Second, blood was evaluated for viraemia at a routine control after both the second and fourth cycle of chemotherapy, when the child presented without infectious complication (“regular control during chemotherapy”). Last, blood was assessed when the patient presented with fever during neutropenia, irrespective whether a bacterial pathogen or another source of infection had been identified (“febrile neutropenia”).

### Virological tests

DNA was extracted from 500 µl plasma sample using Qiasymphony DSP virus/pathogen midi kit (Qiagen GmbH, Hilden, Germany) according to the manufacturer´s instructions. CMV viral load was estimated as previously described^10^. The lower limit of CMV quantification was 200 IU/ml, and positive qualitative results of tests below this limit were indicated as < 200 IU/ml. Extracted DNA was also used for semi-quantitative determination of adenovirus, HSV1/2, HHV6 and EBV-specific DNA^11,12^. According to the results of ring-tests (inter-laboratory tests) to evaluate the reproducibility of virus detection between different laboratories, the limit of quantification was 500 copies/ml for all assays. Copy numbers of the different viruses were estimated using these tests results, whereas positive qualitative results of tests below the limit of quantification were indicated as < 500 copies/ml. Specific primer und probes used are listed in Supplement Table 1.

Commercially available diagnostic testing kits were used to assess specific IgG antibodies against adenovirus (VirClia Adenovirus IgG, Bestbion dx, Cologne, Germany), HSV1 and HSV2 (LIAISON® XL HSV-1 IgG, LIAISON® HSV-2 IgG, DiaSorin, Dietzenbach, Germany), EBV-VCA (LIAISON® XL VCA IgG, DiaSorin), CMV (Architect CMV-IgG, Abbott Diagnostics, Wiesbaden, Germany), and HHV6 (HHV-6 IgG IFT, Euroimmun, Lübeck, Germany). The interpretation of the serological tests followed the manufacturer’s instructions.

As this was an exploratory study and in order to avoid that viral test results will influence a clinical decision regarding antiviral treatment, all serum samples for PCR testing were frozen at − 20 °C and kept until the patient had completed intensive chemotherapy.

### Statistical analysis

Data were analysed using GraphPad Prism (version 5.04; Graph-Pad Software; https://www.graphpad.com/). Data on PCR results in the different populations and time points were reported in a descriptive way. For comparing febrile neutropenic episodes in patients with positive viral PCR to those with negative results of viral PCR, the Mann–Whitney *U* test (for comparison of the median) or Chi-square-test or Fisher’s exact test was used. Only the first episode of reactivation with a specific virus during febrile neutropenia was included in the analysis, as repeated episodes of reactivation with the same virus were not considered to be independent events. All statistical tests were two-sided, and a *P* value of < 0.05 was considered as significant.

### Conference presentation

Part of this work was presented at the Annual Meeting of the German Society of Paediatric Oncology and Hematology, November 10, 2023, Frankfurt, Germany.

### Ethics approval

This study was performed in line with the principles of the Declaration of Helsinki. The study was reviewed and approved by the local Ethical committee of the University of Frankfurt (October 21, 2019; 19/402). All patients and/or caregivers provided written informed consent.

## Results

From August 1, 2020 until July 31, 2022, a total of 79 patients [32 male, 47 female, median age 6 years (range, 0–18)] were included in the study. Thirty-six children were diagnosed with ALL, 16 with AML, 14 with NHL, 3 with Ewing sarcoma, and 10 with neuroblastoma (Table 1). Thirty-one (39.7%) of the paediatric patients had detectable CMV-IgG at the time of the diagnosis of the malignancy (CMV-IgG available in 78 patients), 29 (38.6%) had detectable HSV1/2-IgG (available in 75 patients), and 31 (41.3%) had EBV-IgG (available in 75 patients), respectively. A total of 362 blood samples were analysed, 72 from the time at diagnosis [8 (11.1%) with a positive PCR result], 118 during a regular control after chemotherapy [12 (11%) with a positive PCR result; 59 each after the second and the fourth course of chemotherapy, respectively], and 159 during a febrile neutropenic episode [14 (8.8%) with a positive PCR result] (Table 1). Overall, 34 of the 362 blood samples (9.3%) were positive for one of the viruses tested, namely 12 for CMV (3.3%; viral load (range) < 200–440 IU/ml), 10 for HHV6 (2.7%; viral load (range) < 500–600,000 copies/ml), 8 for HSV1/2 (2.2%; viral load (range) < 500–1000 copies/ml), 3 for EBV (0.8%; viral load (range) < 500–5000 copies/ml) and 1 for adenovirus (0.3%; viral load < 500 copies/ml), respectively (Table 1). During the study, no viral testing of the serum was performed in any patient as clinically indicated evaluation outside the study.

**Table 1:** Patients´ characteristics and number of positive PCR results at the time of diagnosis, during a regular control after chemotherapy and during an episode of febrile neutropenia.

Malignancy	Number of patients	Age [years; median (range)]	Sex (female/male)	Total number of samples	Number and viral load* of positive samples at diagnosis of at regular during febrile malignancy control neutropenia		
ALL	36	6 (1–17)	11/25	185	2 (EBV 1 (< 500), HHV6 1 (600,000))	6 (HSV1/2 4 (4x < 500), HHV6 1 (13,000), CMV1 (< 200))	7 (CMV 2 (2x < 200); HHV6 2 (18.000/9.000); HSV1/2 2 (< 500/700); adenovirus 1 (< 500))
AML	16	4 (8–18)	7/9	75	–	1 (HHV6 1 (< 500))	3 (HHV6 2 (< 500/ < 500), CMV 1 (< 200))
NHL	14	11 (2–18)	5/9	44	4 (HSV1/2 1 (1.000), CMV 1 (440), HHV6 1 (150,000), EBV 1 (5000))	2 (HHV6 2 (14.000/14.000))	–
Neuroblastoma	10	2 (0–5)	7/3	48	2 (CMV 1 (< 200), EBV 1 (360))	3 (CMV 3 (2x < 200/290))	4 (CMV 3 (3x < 200), HSV1/2 1 (< 500))
Ewing	3	15 (11–18)	2/1	10	–	–	–

*ALL* acute lymphoblastic leukemia, *AML* acute myeloid leukemia, *NHL* non-Hodgkin lymphoma, *EBV* Epstein-Barr virus, *HHV6* human herpesvirus-6; *CMV* cytomegalovirus, *HSV1/2* herpes simplex virus ½.

*viral load for CMV expressed in IU/ml, for all other viruses in copies/ml.

Fifty-seven patients (72.2%) did not have any positive PCR result during the study period. Out of these, 17 (29.8%), 21 (36.8%) and 22 (38.5%) patients had a positive test result for specific IgG against CMV, HSV1/2 and EBV, respectively, at the time of the diagnosis of the malignancy. Twenty-two patients (27.8%) had at least one positive PCR result during the study period, with positive results for specific IgG against CMV, HSV1/2 and EBV in 9 (40.9%), 7 (31.8%) and 9 (40.9%) of the patients, respectively (no significant difference between patients with specific IgGs who had or did not have a positive PCR during the study period). In 9 patients with ALL (25% of patients with ALL), 3 with AML (18.8%), 5 with NHL (35.7%), and 5 with neuroblastoma (50%) a positive sample for viraemia was found during the study period, whereas none of the patients with Ewing sarcoma had a positive PCR result. Thirteen of the patients had one single positive PCR result during the study period (4 patients prior to therapy (CMV n = 2, HSV1/2 1, EBV 1), 6 patients at a regular control during chemotherapy (HSV1/2 4, CMV 1 und HHV6 1), and 3 patients during febrile neutropenia (CMV 2, HSV1/2 1)]. In contrast, PCR testing revealed more than one positive result during the study period in 9 patients: a positive PCR result with the same virus was seen in 5 patients (three patients with HHV6, two with CMV), and different viruses were detected in 4 patients.

In a total of ten patients, at least one positive PCR was observed during an episode of febrile neutropenia (Table 2). One febrile neutropenic episode with viral detection was observed in six patients (#2. #4, #7—#10), whereas in three patients (#1, #5 and #6), the same virus (HHV6, n = 2, CMV, n = 1) was detected in two distinct episodes of febrile neutropenia, and in one patient (#3), different viruses were found in two febrile neutropenic episodes (HSV1/2 and CMV). The underlying diagnoses of patients with at last one positive viral PCR finding during febrile neutropenia were ALL (n = 5), AML (n = 2), and neuroblastoma (n = 3) (Table 2). The median number of neutrophils and lymphocytes was 70/µl, (range 0–500) and 310/µl (range 60–6780), respectively. Mucositis > II^o^ was present in three patients (2 of them had 2 FN episodes with a positive viral test) (patients #1; 3; 6), one patient had both adenovirus in blood and stool (#8), and *Clostridioides difficile* was found in 3 patients (#3–5). None of the patients with viraemia was referred to the ICU, none received systemic antiviral treatment.

**Table 2:** Patients with viraemia during an episode of neutropenia.

Patient number and malignancy (risk group)	Age (years)/sex	CMV IgG*	HSV 1 IgG*	EBV VCA IgG*	Number of neutrophils / lymphocytes at detection of virus (per µl)	Virus detected (viral load*)	Days with neutropenia /lymphopenia	Days with fever	Mucositis > II^o^	Diarrhea
#1 NB (MR)	2, f	pos	neg	neg	10 / 170 0 / 180	CMV (< 200) CMV (< 200)	10 / 9 9 / 8	6 5	yes yes	no no
#2 AML (HR)	1, m	pos	n.d	neg	0 / 70	CMV (< 200)	30 / 18	1	no	no
#3 ALL (HR)	16, m	pos	pos	pos	500 / 440 40 / 50	CMV (< 200) HSV1/2 (< 500)	4 / 0 13 / 16	2 2	yes yes	yes no
#4 NB (MR)	1, f	pos	neg	neg	100 / 320	CMV (< 200)	15 / 0	1	no	yes
#5 ALL (HR)	15, m	pos	neg	pos	300 / 310 330 / 720	HHV6 (18,000) HHV6 (9000)	8 / 6 12 / 0	3 1	no no	yes no
#6 AML (HR)	8, m	pos	pos	pos	90 / 60 50 / 100	HHV6 (< 500) HHV6 (< 500)	55 / 11 42 / 25	8 2	yes yes	no no
#7 NB (HR)	6, m	neg	neg	pos	40 / 820	HSV1/2 (< 500)	7 / 0	2	no	no
#8 ALL (MR)	1, m	neg	neg	neg	480 / 6780	ADV (< 500)	2 / 0	7	no	yes
#9 ALL (HR)	10, f	neg	pos	pos	310 / 510	HSV1/2 (700)	33 / 2	6	no	no
#10 ALL (HR)	6, m	pos	neg	neg	10 / 690	CMV (< 200)	32 / 0	1	no	no

*NB* neuroblastoma, *AML* acute myeloid leukemia, *ALL* acute lymphoblastic leukemia, *MR* intermediate risk, *HR* high risk, *f* female, *m* male, *neg* negative, *pos* positive, *n.d.* not done, *EBV* Epstein-Barr virus, *HHV6* human herpesvirus-6, *CMV* cytomegalovirus, *HSV* herpes simplex virus, *IgG* immunoglobulin G.

*prior to diagnosis of malignancy.

*viral load for CMV expressed in IU/ml, for all other viruses in copies/ml.

In order to assess the clinical relevance of viraemia in febrile neutropenic patients, we compared the first episode of a specific viral infection in patients with at least one positive PCR result during febrile neutropenia (n = 11) with febrile episodes of patients without positive PCR result (matched-pair comparison regarding underlying disease, treatment, sex and age; each febrile neutropenic episode with viraemia was compared with 2 matched episodes without positive PCR). There were no significant differences in days of fever [median 2 (range 1–8) versus 2 (range 1–8)], days of neutropenia [median 13 (range 2–55) versus 17 (range 2–60)], days of lymphopenia [median 2 (range 0–18) versus 5 (0–22)], mucositis > II^o^ (4/11 versus 8/24), diarrhea (4/11 versus 2/24), and ICU admission (none each).

## Discussion

While infections with viruses such as CMV, HSV or adenovirus are a common and often severe problem in children after HCT and mandate the institution of antiviral therapy in many patients, the frequency and clinical significance of a positive viral PCR in the blood of children receiving conventional chemotherapy is relatively unclear. In our prospective monocentre study, a total of 9.3% of the 362 blood samples were positive for one of the viruses tested, namely in 3.3% for CMV, 2.7% for HHV6, 2.2% for HSV1/2, 0.8% for EBV and 0.3% for adenovirus, respectively. Overall, 27.8% of the patients had at least one positive PCR finding during intensive chemotherapy. A study in 77 children receiving chemotherapy for a variety of underlying malignancies reported similar findings for CMV and EBV with 3.4% and 0.5% of positive samples, respectively, but also observed that 9.3% of samples were positive for HHV6, which is considerably higher compared to our results^13^. Published data demonstrate a wide variation in the prevalence of viraemia in patients treated for cancer, which, for example, ranges from 2.5% to 52% for CMV or from 5.9% to 26.3% for HHV6^14–16^. These differences might be explained by several factors, such as by differences in the age range of tested patients, different methods of virus detection and different thresholds of positivity^14,16–18^. Due to different sampling strategies (e.g., screening every two weeks throughout intensive chemotherapy versus sample collection only during prolonged febrile neutropenia) resulting in a wide variation of the number of samples (between 141 and 1,223), a meaningful comparison of the studies regarding the percentage of patients with viraemia cannot be made^13,15^. Corroborating our data, no major impact of the underlying malignancy on the incidence of viraemia had been observed in other studies^13,16^. Our observation that there were no differences in overall frequencies of viral detection between patients who were seropositive or seronegative at diagnosis is different from previous studies and might be explained by differences in the prevalence of seropositivity and by the small sample size^15,17^. We did not find a significant difference of the frequency of viraemia between test results prior to chemotherapy, test results during a routine control and those during an episode with febrile neutropenia, which were 11.1%, 11.0% and 8.8%, respectively. Similar results were observed by Goldfarb et al. with 11.5% and 9% of positive results in acute and routine visits, respectively^13^, whereas others reported that the detection of HHV6 in blood doubled from initial assessment to testing during a febrile episode (17.4% to 37.1%)^18^. Notably, one study reported that the majority of CMV reactivation in ALL patients occurred during maintenance therapy, a time period which was not included in our analysis^19^. This observation can be explained by the fact that maintenance therapy in ALL consists of daily 6-mercaptopurine and weekly methotrexate, which leads to prolonged lymphopenia, and low lymphocyte counts have been described as a risk factor for CMV reactivation^16^.

Severe HHV6 infections have been observed in up to 6% of cancer patients during treatment with cytotoxic chemotherapy, and HHV6, in particular during a coinfection with CMV, significantly increases the risk for serious clinical complications, such as bacterial and fungal infections^16,18^. Importantly, also fatal events due to virus infection have been described in children receiving therapy for cancer^20^. On the other hand, it is currently unclear whether viraemia in febrile neutropenic children who are otherwise asymptomatic has clinical consequences and patients benefit from antiviral therapy, even in case of high copy numbers. For example, in one study, 7 asymptomatic children suffering from ALL revealed high level of CMV viraemia^15^. According to the physician´s discretion, four of them were treated with intravenous ganciclovir with or without subsequent oral valganciclovir, whereas three patients did not receive antiviral treatment. Whether treated or not, all patients remained well during the entire study and no end-organ diseases were detected. In our analysis, we did not observe any severe clinical complications due to virus infection, and in none of the patients, organ involvement (e.g., retinitis, pneumonia, hepatopathy) occurred. In addition, none of the patients received preemptive antiviral therapy. However, we observed only low levels of viraemia in the patients, which is similar to the results of other studies^14^. In addition, in comparison with case-matched controls without viraemia we found that children receiving chemotherapy and having viraemia do not experience more days of neutropenia or of lymphopenia, and do not suffer from longer duration of fever, higher grade of mucositis, higher incidence of diarrhea or ICU admission than children without positive PCR results. Corroborating our data, others did not find an association between HHV6 viraemia and individual acute clinical complications, and therefore, clinical events may be misattributed to the virus^13^. Based on the current data and the fact that many antiviral drugs are associated with severe potential adverse events, we do not preemptively treat even high level viraemia in asymptomatic paediatric patients receiving chemotherapy for an underlying malignancy, which is in contrast to the setting of HCT^21^. In addition, we do not screen febrile neutropenic, but otherwise asymptomatic patients for viraemia, which has been suggested by some experts^14,19^.

We recognise that our study has some important limitations. Although this is one of the largest prospective studies in children, it is a monocentre approach which does not allow to generalise the findings. In addition, due to the irregularity of sampling intervals, we might have missed positive findings, which could be associated with clinical complications. Last, we did not analyse the time of maintenance therapy, which might have higher incidence rates of viraemia^15,19^. On the other hand, our study is unique as all virological testing of blood samples was performed after the end of intensive chemotherapy of a specific patient, and therefore, data and in particular treatment decisions are unbiased.

In conclusion, our data need to be validated in other centres and with other therapy protocols, but our results indicate that viraemia in paediatric cancer patients receiving intensive chemotherapy generally does not have a major clinical impact, and may help in the decision regarding the indication of routine evaluation for viraemia in febrile neutropenic, but otherwise asymptomatic children.

### Supplementary Information

Supplementary Information.

## Data Availability

All data and material related to this study are available from the corresponding author on request (Thomas_Lehrnbecher@yahoo.com).
