# Supplementary material for: Frequency and clinical impact of viraemia in paediatric patients undergoing therapy for cancer
Source: Sci Rep. 2024 Jun 27;14:14867. doi: 10.1038/s41598-024-65641-w (PMC11211494; doi:10.1038/s41598-024-65641-w)
Supplement: Supplementary file 1 — Supplementary Information. [file 41598_2024_65641_MOESM1_ESM.docx]

**Supplementary Table 1:** Primer and probes used for semi-quantitative assessment of specific viruses

| **Virus** | **Primer and probes** |
| --- | --- |
|  |  |
| *Adenovirus* | F-Primer gCCAcggTggggTTTCTAAACTT |
|  | R-Primer gCCCCAgTggTCTTACATgCACATC |
|  | Probe VIC-TgCACCAgACCCgggCTCAggTACTCCgA-TAMRA |
|  |  |
| *HSV1/2* | F Primer AggAgCCCgTCCCCTTTC |
|  | R Primer gCCCCgCgCCTAAAgT |
|  | Probe VIC-CggCTCCACgAggCCCTg-TAMRA |
|  |  |
| *EBV (BNRF1 gene)* | F Primer CCAgTgCTgTgATCgAgCATC |
|  | R Primer CTgCTgACAAACTgCTgCATTC |
|  | Probe VIC-TCTgCTgTTgTTTCTgTCTCACCTACgg-TAMRA |
|  |  |
| *HHV6 Region U67* | F Primer CgC TAg gTT gAg RAT gAT CgA |
|  | R Primer CAA AgC CAA ATT ATC CAg AgC g |
|  | Probe: VIC-CAC CAg ACg TCA CAC CCg AAg gAA T –TAMRA |

**Supplementary Table 2:** Results of initial serology and PCR results at the time of diagnosis, during a regular control after chemotherapy and during an episode of febrile neutropenia [total sample number (positive result/negative result)]

| **Virus** | **Initial serology (IgG)** | **PCR**  **at diagnosis of malignancy** | **PCR at regular control** | | **PCR during febrile neutropenia** | **Total number of positive results** |
| --- | --- | --- | --- | --- | --- | --- |
|  |  |  | **After second chemotherapy** | **After fourth chemotherapy** |  |  |
| **CMV** | 78 (31/47) | 72 (2/70) | 59 (3/56) | 59 (1/58) | 159 (6/153) | 12 |
| **HSV1/2** | 75 (29/46) | 72 (1/71) | 59 (4/55) | 59 (0/59) | 159 (3/156) | 8 |
| **EBV** | 75 (31/44) | 72 (3/69) | 59 (0/59) | 59 (0/59) | 159 (0/159) | 3 |
| **HHV6** | 10 (9/1) | 72 (2/70) | 59 (2/57) | 59 (2/57) | 159 (4/155) | 10 |
| **Adeno** | n.d. | 72 (0/72) | 59 (0/59) | 59 (0/59) | 159 (1/158) | 1 |

CMV cytomegalovirus; HSV1/2 herpes simplex virus 1/2; EBV Epstein-Barr virus; HHV6 human herpesvirus-6; Adeno adenovirus; n.d. not done
